# In vitro synergy of isavuconazole in combination with colistin against *Candida auris*

**DOI:** 10.1038/s41598-020-78588-5

**Published:** 2020-12-08

**Authors:** Patrick Schwarz, Anne-Laure Bidaud, Eric Dannaoui

**Affiliations:** 1 Department of Internal Medicine, Respiratory and Critical Care Medicine, University Hospital Marburg, Baldingerstraße, 35043 Marburg, Germany; 2 Center for Invasive Mycoses and Antifungals, Philipps University Marburg, 35037 Marburg, Germany; 3 Unité de Parasitologie-Mycologie, Hôpital Européen Georges Pompidou, AP-HP, Faculté de Médecine, Université de Paris, 75015 Paris, France; 4 EA 7380 Dynamyc, Université Paris-Est Créteil, Ecole Nationale Vétérinaire D’Alfort, USC Anses, 94010 Maisons-Alfort, France

**Keywords:** Microbiology, Antimicrobials, Antibiotics, Antifungal agents, Antimicrobial resistance

## Abstract

The in vitro interactions of isavuconazole with colistin were evaluated against 15 clinical *Candida auris* isolates by a microdilution checkerboard technique based on the EUCAST reference method for antifungal susceptibility testing and by agar diffusion using isavuconazole gradient concentration strips with or without colistin incorporated RPMI agar. Interpretation of the checkerboard results was done by the fractional inhibitory concentration index and by response surface analysis based on the Bliss model. By checkerboard, combination was synergistic for 93% of the isolates when interpretation of the data was done by fractional inhibitory concentration index, and for 80% of the isolates by response surface analysis interpretation. By agar diffusion test, although all MICs in combination decreased compared to isavuconazole alone, only 13% of the isolates met the definition of synergy. Essential agreement of EUCAST and gradient concentration strip MICs at +/− 2 log_2_ dilutions was 93.3%. Antagonistic interactions were never observed for any technique or interpretation model used.

## Introduction

*Candida auris* is an emerging, multidrug-resistant, fungal pathogen, responsible for invasive hospital-acquired infections^1–3^. The yeast was first isolated from an ear sample in Japan and described in 2009^4^. Initially supposed to be a rare pathogen, the incidence of this *Candida* species has increased and cases have been reported in several countries across five continents^5^. The emergence of *C. auris* is alarming, as the species has the potential to exhibit or to develop multidrug-resistance to antifungals^6,7^. *C. auris* is associated with a cumulative mortality rate of 33% in all reported cases until 2018 despite antifungal treatment^8^. Antifungal combination may be an interesting therapeutic approach to improve the prognosis of the patients. Indeed, combination of two molecules may reduce toxicity, decrease antifungal dosages, improve the pharmacokinetics of one or both drugs, and more importantly achieve synergistic interactions^9^. Nevertheless, only few studies have evaluated in vitro combinations against *C. auris*^10–13^. First of all, the combination of micafungin with voriconazole was synergistic against all tested isolates^12^. Another more recent study evaluated the combination of flucytosine with amphotericin B, voriconazole or micafungin against 15 isolates and showed no antagonism^10^. Antifungal combinations including flucytosine were also evaluated against *C. auris* isolates from a New York outbreak^13^. Combinations of antifungals with non-antifungal drugs such as colistin have also been tested^11^. Colistin belongs to the family of polymyxins, from the group of polymyxins E. It targets the external membrane of the gram negative bacteria, more precisely lipid A, a lipopolysaccharide^14^. This leads to an alteration of the external membrane, resulting in an increased membrane permeability, finally leading to cell death^15^. In *Candida albicans* and *Rhizopus arrhizus*, it has been shown that colistin damages the cytoplasmic membrane^16,17^. This mechanism of colistin could favour the penetration of antifungals, which would make the antibiotic an interesting partner to use in combination therapy for the treatment of fungal infections. Colistin has been tested in vitro against yeasts^11,17–21^, and filamentous fungi^16,17,19,20^, and in vivo in animal models of invasive candidiasis^18,21^, and mucormycosis^16^ with various activities and efficacies. Isavuconazole is a new broad spectrum azole recommended for treatment of invasive aspergillosis and mucormycosis^22^, but has also shown in vitro activity against several yeast species including *C. auris*^23,24^. Therefore, combination of colistin with isavuconazole may be of interest against *C. auris*. The objective of the present study was to evaluate the in vitro interaction of isavuconazole with colistin against a collection of clinical *C. auris* isolates.

## Results

The interactions between isavuconazole and colistin evaluated by checkerboard (interpreted by FICI or by response surface analysis) and by agar diffusion assay against the 15 *C. auris* isolates are presented in Table 1. Percentages of the interactions of the different techniques and interpretation models are presented in Table 2.

**Table 1:** Interaction of isavuconazole with colistin against *Candida auris* by checkerboard and interpretation by fractional inhibitory concentration index, response surface analysis and by agar diffusion.

Species	Collection number	Checkerboard MICs (µg/mL)			FICI	INTPN	Response surface analysis		Agar diffusion assay^a^ MICs (µg/mL)		
		ISA	COL	ISA/COL			SUM-SYM-ANT	INTPN	ISA	ISA + COL	INTPN
*C. auris*	CBS 10913	0.004	128	0.001/32	0.5	SYN	34.8	SYN	0.006	0.003	IND
*C. auris*	CBS 12372^b^	1	128	0.25/32	0.5	SYN	− 8.94	IND	1	0.5	IND
*C. auris*	CBS 12373^c^	0.25	128	0.125/1	0.5078	IND	25.05	SYN	3	0.5	SYN
*C. auris*	CBS 12766	0.25	128	0.06/8	0.3125	SYN	31.43	SYN	0.25	0.19	IND
*C. auris*	CBS 12767	0.25	128	0.06/8	0.3125	SYN	23.91	IND	0.5	0.25	IND
*C. auris*	CBS 12768	0.25	128	0.06/8	0.3125	SYN	25.13	SYN	0.38	0.25	IND
*C. auris*	CBS 12769	0.25	128	0.06/8	0.3125	SYN	23.38	IND	0.75	0.25	IND
*C. auris*	CBS 12770	0.5	128	0.125/8	0.3125	SYN	25.49	SYN	1	0.5	IND
*C. auris*	CBS 12771	0.25	128	0.06/32	0.5	SYN	28.04	SYN	1	0.25	SYN
*C. auris*	CBS 12772	0.25	128	0.06/16	0.375	SYN	44.93	SYN	0.5	0.25	IND
*C. auris*	CBS 12773	0.125	128	0.03/32	0.5	SYN	37.50	SYN	0.5	0.19	IND
*C. auris*	CBS 12774	0.25	128	0.06/16	0.375	SYN	38.7	SYN	0.75	0.38	IND
*C. auris*	CBS 12775	0.25	128	0.06/16	0.375	SYN	55.42	SYN	0.5	0.38	IND
*C. auris*	CBS 12776	0.25	128	0.06/16	0.375	SYN	58.02	SYN	0.38	0.25	IND
*C. auris*	CBS 12777	0.25	128	0.06/32	0.5	SYN	42.71	SYN	0.5	0.38	IND

FICI, fractional inhibitory concentration index; INTPN, interpretation; SYN, synergy; IND, no interaction; ISA, isavuconazole; COL, colistin; CBS, Westerdijk Fungal Biodiversity Institute. ^a^Colistin alone at 64 µg/mL incorporated in the RMPI agar did not inhibit the growth of any of the isolates. ^b^Other collection number: KCTC 17809. ^c^Other collection number: KCTC 17810.

**Table 2:** Summary of interactions of isavuconazole with colistin against 15 *Candida auris* isolates by checkerboard and interpretation by fractional inhibitory concentration index, response surface analysis and by agar diffusion.

Technique (interpretation)	% of isolates with the following interaction		
	Synergy	No interaction	Antagonism
Checkerboard (FICI)	93	7	0
Checkerboard (RSA)	80	20	0
Agar diffusion	13	87	0

FICI, fractional inhibitory concentration index; RSA, response surface analysis.

By microbroth dilution method, the 15 isolates exhibited MICs for isavuconazole alone ranging from 0.004 to 1 µg/mL with a MIC50, MIC90, and geometric mean MIC of 0.25, 0.5, and 0.21 µg/mL, respectively (Table 1). Between the experiments, the isavuconazole MICs were within +/− 1 log_2_ dilutions in 100% of the cases (data not shown). Colistin alone did not exhibit in vitro activity alone at the concentrations used for the experiment. MICs for all isolates were > 64 µg/mL. By checkerboard and interpretation by fractional inhibitory concentration index (FICI), the interaction of isavuconazole with colistin was synergistic for 93% of the isolates with FICIs ranging from 0.3125 to 0.5 with a mean FICI of 0.4.

Analysis of the checkerboard data by the response surface approach led to similar results. For the combination of isavuconazole with colistin the SUM-SYN-ANT metric ranged from − 8.94 to 58.02 (Table 1), with a mean of 32.37. Overall synergy was obtained for 80% of the isolates (Table 2).

By agar diffusion assay MICs of isavuconazole were slightly higher as by checkerboard (*p* = 0.013) and ranged from 0.006 to 3 µg/mL with a MIC50, MIC90, and a geometric mean MIC of 0.5, 1, and 0.47 µg/mL, respectively. Nevertheless, there was a good correlation between EUCAST and gradient concentration strip MICs with an essential agreement, at +/− 2 log_2_ dilutions, of 93.3%. Growth on RPMI agar was not inhibited by incorporation of 64 µg/mL of colistin for all isolates. Although all MICs in combination were lower than MICs of isavuconazole alone, only two isolates met the definition of synergy of a decrease ≥ 2 log_2_ dilutions compared to isavuconazole alone. Nevertheless, a decrease of 1 log_2_ was seen for 6 additional isolates and a decrease of 1.5 log_2_ for 2 further isolates.

**Figure 1:**
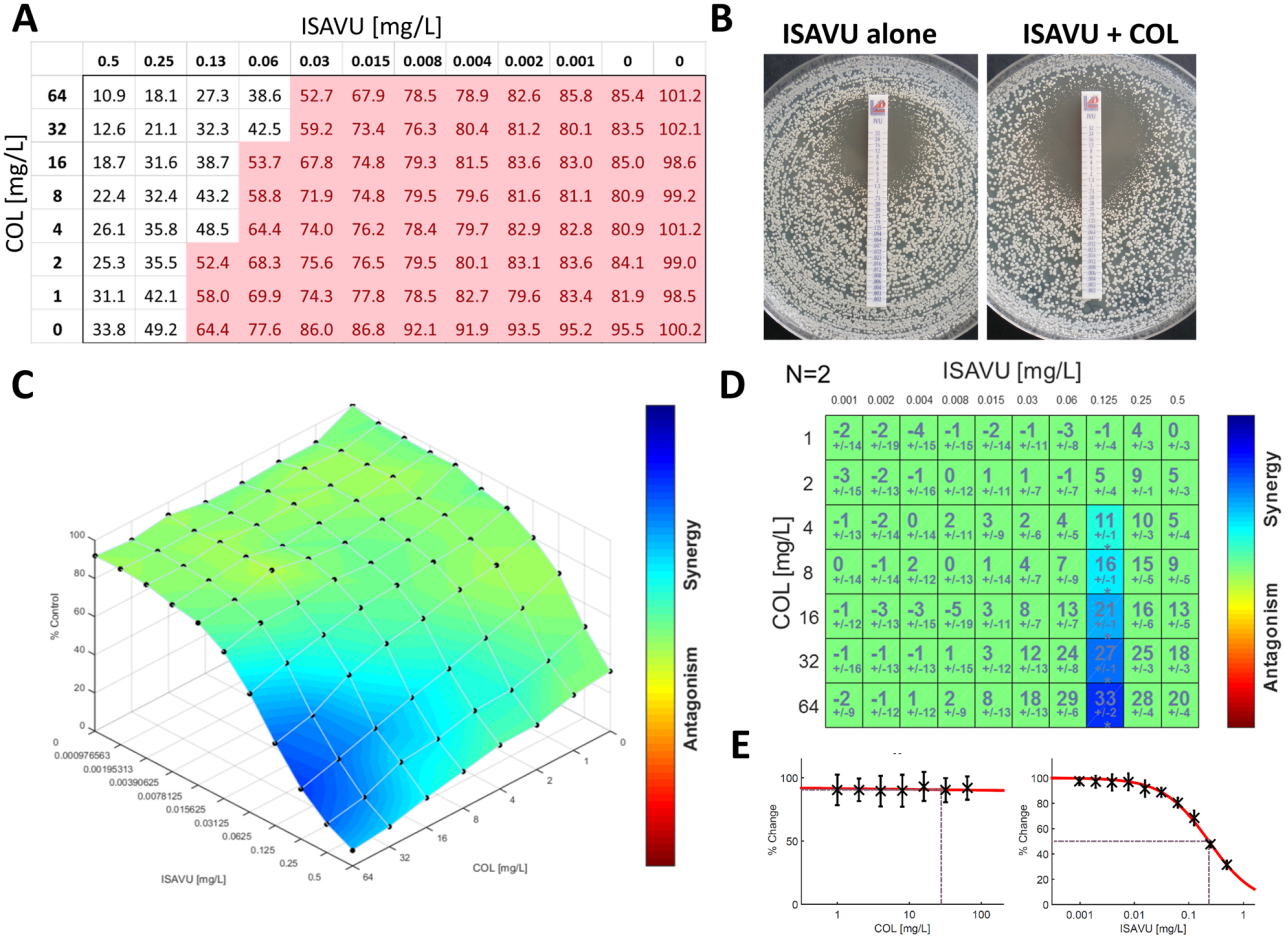
Comparison of the in vitro techniques and interpretation models for the synergistic interaction between isavuconazole (ISAVU) and colistin (COL) against *C. auris* isolate CBS 12771. (**A**) Percentage of growth compared to the growth control on the 96 wells microplate; (**B**) Agar diffusion assay: left side, isavuconazole alone; right side isavuconazole + colistin at 64 µg/mL incorporated in the agar; (**C**) Response surface analysis, based on the Bliss model, showing the mapping of the synergy levels on the experimental combination dose–response surface; (**D**) Synergy distribution, in matrix format, derived from the combination dose–response and the reference dose–response; (**E**) Response curves of the drugs alone. Panels C, D, and E were generated with the software Combenefit, version 2.021^38^.

**Figure 2:**
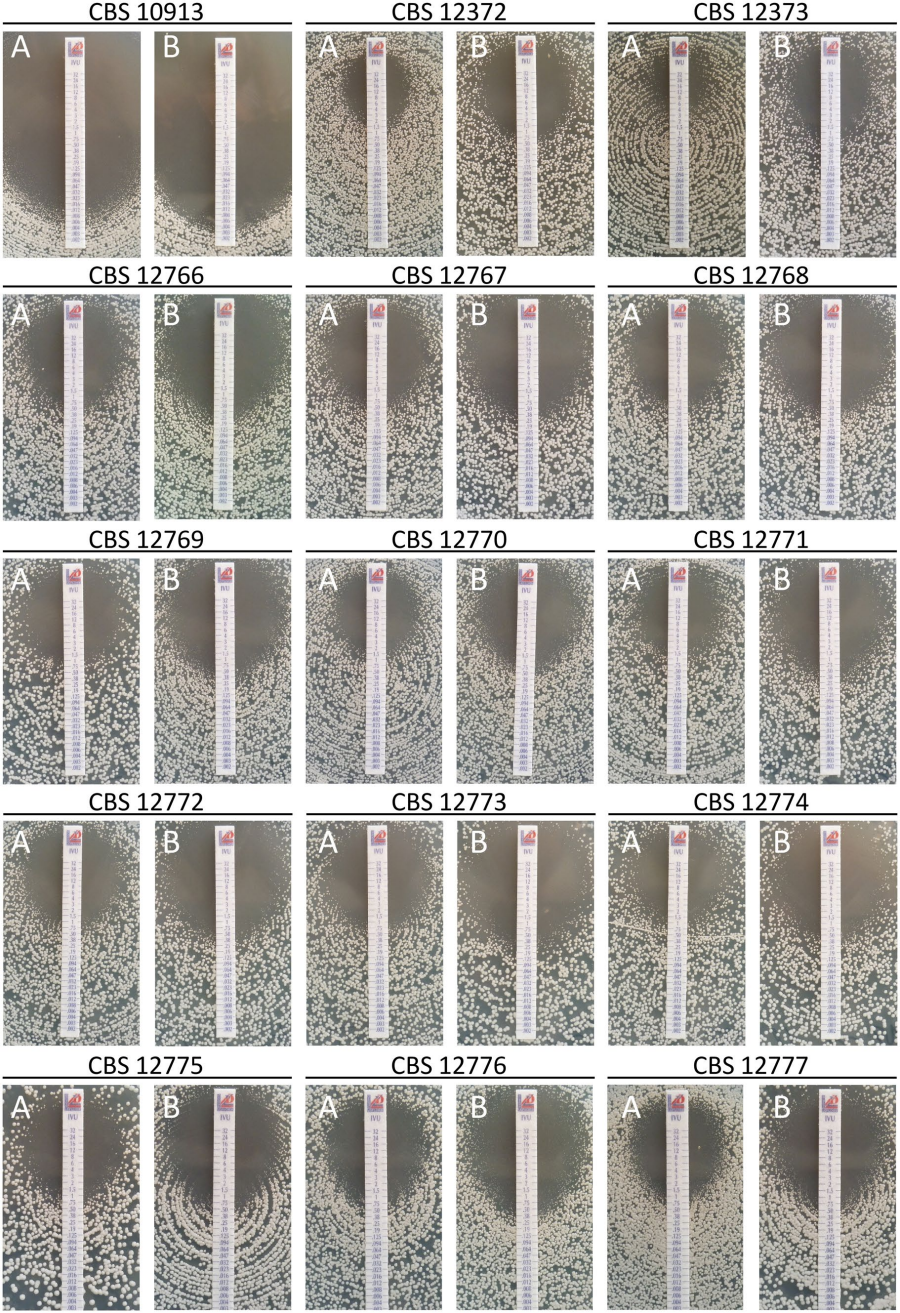
Agar diffusion assay for the 15 isolates. (**A**) Panels: isavuconazole alone; (**B**) panels: isavuconazole + colistin at 64 µg/mL incorporated in the agar. Colistin alone at 64 µg/mL incorporated in the agar did not inhibit the growth of any of the isolates (pictures not shown).

Antagonistic interactions were never observed whatever technique or method of analysis used. Figure 1 shows the synergistic interaction of isavuconazole in combination with colistin for isolate CBS 12771 by the different in vitro techniques and interpretation models used in this study. Figure 2 shows the interaction of isavuconazole in combination with colistin against all 15 isolates by agar diffusion assay.

## Discussion

Recently, the appearance of coronavirus disease 2019 (COVID-19), caused by severe acute respiratory syndrome coronavirus 2 (SARS-CoV-2)^25^, led to a global health crisis, since there were only symptomatic treatment options available^26^. Drugs designated for other diseases were repurposed for COVID-19 to overcome this therapeutic issue. Interventions against SARS-CoV-2 included prophylaxis to augment epithelial defence (e.g. angiotensin II receptor antagonists), reduction of viral load (e.g. remdesivir), tempering inflammatory signalling and injury (e.g. dexamethasone), and inhibitors targeting the molecular mediators of the maladaptive COVID-19 immune response (e.g. IL-6)^27^. There are three major advantages of drug repurposing: first the use of de‐risked compounds, second the potentially lower overall development costs, and third shorter development timelines^28^. Already prior to the COVID-19 pandemic repurposing of drugs had become trendy. Repurposed drugs have shown activity against bacteria^29^, yeasts^29,30^, and filamentous fungi^31–34^. Colistin is a drug of last resort with activity against multidrug-resistant gram-negative bacteria such as *Pseudomonas aeruginosa*, *Klebsiella pneumoniae*, or *Acinetobacter baumanii*^35^; but colistin has also shown to damage the cell membrane of *C. albicans*^17^, which could make it an interesting partner in combination therapy against multidrug-resistant *C. auris* infections. Indeed, resistance to all four major classes of antifungals (polyenes, azoles, pyrimidines, and echinocandins) has been reported in clinical isolates of *C. auris*. Therefore, combination therapy may be an interesting option compared to monotherapies for management of antifungal-resistant *C. auris* infections.

One of the problems of assessing antifungal combinations by the FICI is the choice of the endpoint. Fifty percent of inhibition is recommended for yeasts and azoles for the determination of the MICs of antifungals alone^36^. Nevertheless, for combination studies, there is no consensus about which endpoint should be used, particularly when one of the partner drugs is not an antifungal, which is the case in this study. In combination studies, the major point is to look for relative growth when antifungals are tested alone and in combination. In some interaction models (such as Prichard model based on Bliss independence theory^37^) there is no endpoint at all, and the interaction is calculated only based on the percentage of growth at different concentrations. We additionally used an alternative model not dependent of an endpoint (surface-response based on the Bliss model^38^) to overcome the limitations of the FICI model. This model has already been used successfully against medically important *Candida* species^39^, including *C. auris*^10,11^.

In vitro synergy between colistin and antifungals has been reported for yeasts^11,17,18,20,21^, and filamentous fungi^17,20^. However, absence of synergy^11,17,19,21^, and even antagonism have also been reported^19^. In our study, we found in vitro synergy in the combination of isavuconazole with colistin by checkerboard, and in the interpretation of the data by two radically different approaches (FICI or response surface analysis). By agar diffusion assay no interaction of the combination was seen. This may be evidence that the synergy of the combination is weak. Nevertheless, the MICs in combination were reduced for all isolates compared to isavuconazole alone. Another evidence for a weak synergy of the combination is the relatively high mean FICI of 0.4. Additionally, the mean SUM-SYN-ANT of 32.37 was relatively low, compared to another study where a strong synergy for the combination of colistin with caspofungin with a mean SUM-SYN-ANT of 122.3 was found^11^. The only other study which evaluated the interaction of colistin with antifungals against *C. auris* demonstrated that colistin in combination with caspofungin was synergistic, and that combination with micafungin was not antagonistic^11^. In the same study, colistin showed no in vitro activity when used alone. Our results are in concordance to this study. Isavuconazole alone exhibited in vitro activity against *C. auris* by broth microdilution and by gradient concentration strips with MICs in the same range and an essential agreement between the two techniques as previously published^24^. Combinations of colistin with other antifungals against *Candida* species were mainly tested against *C. albicans*. Colistin combined with caspofungin was synergistic against *C. albicans, *in vitro and in vivo in a *Galleria mellonella* model of invasive candidiasis^18^. Combinations of colistin with echinocandins have also been tested against *C. albicans* and other *Candida* species in another study. Combinations exhibited synergy, but unfortunately, only few isolates were tested^21^. In an another study, the combination of colistin with the polyene amphotericin B or the azole itraconazole also showed synergistic interactions against one *C. albicans* isolate^17^.

In our study, broth microdilution MICs of colistin in combination ranged from 1 to 32 µg/mL. In patients with cystic fibrosis receiving prolonged colistin therapy, peak serum levels of 13 to 32 µg/mL were measured^40^, which would be sufficient to be effective against our isolates; but it has to be noted that the use of colistin in critically ill patients is limited due to its nephrotoxicity^41^. In critically ill patients peak levels of 0.5 to 9.4 µg/mL were reported^42^. Nevertheless, in vivo synergistic interactions could be present at lower concentrations than those tested in vitro. Indeed, one study evaluated the in vitro interaction of tacrolimus with amphotericin B or fluconazole against *Cryptococcus neoformans* by checkerboard, and synergy was obtained for both combinations. Despite lower achievable tacrolimus serum concentrations than the concentrations used in the microplates, the outcome of solid organ transplant recipients with cryptococcosis receiving tacrolimus long-term therapy and amphotericin B or fluconazole, was significantly better than the outcome of patients receiving only amphotericin B or fluconazole^43^.

In summary, colistin enhances the in vitro activity of isavuconazole against clinical *C. auris* isolates. These results warrant further in vivo experiments.

## Materials and methods

### Isolates

A panel of 15 clinical *C. auris* isolates from the collection of Westerdijk Fungal Biodiversity Institute (Utrecht, The Netherlands) was used for the experiments. All isolates have been identified by molecular biology previously^4,44–46^. The isolates were subcultured from frozen stocks on Sabouraud dextrose agar slants containing chloramphenicol and gentamycin (Bio-Rad Laboratories, Feldkirchen, Germany) for 24 h at 35 °C to ensure purity and viability. The reference strains *Candida krusei* ATCC 6258 and *Candida parapsilosis* ATCC 22,019 were included as quality controls^36^.

### Medium preparation

As a test medium, Roswell Park Memorial Institute 1640 (RPMI) medium (with l-glutamine, with pH indicator, without bicarbonate) (Merck, Darmstadt, Germany) supplemented with d-glucose to a final concentration of 2%, buffered with 3-(N-morpholino) propanesulfonic acid (MOPS) (Merck) at a final concentration of 0.165 mol/L, and adjusted to pH 7.0 with 1 M sodium hydroxide, was used. To allow two-fold dilutions, and the preparation of RPMI petri dishes, the medium was prepared in double strength. After preparation, the medium was sterilized by vacuum filtration through a 0.22 µm pore size filter (Merck)^36^.

### Drugs and microplate preparation

The combination was tested using the EUCAST guidelines for antifungal susceptibility testing of yeasts^36^, with modifications for a broth microdilution checkerboard procedure. Experiments were performed using Nunclon delta surface 96-wells microtiter plates for adherent cells (Thermo Fisher Scientific, Darmstadt, Germany). The included drugs were isavuconazole (Pfizer, Berlin, Germany) and colistin (Merck). The stock solutions of isavuconazole (3200 µg/mL) and colistin (12,800 µg/mL) were prepared in DMSO and sterile, distilled water, respectively. The drug dilutions were performed at four times the final concentration in double strength RPMI medium. The combination was studied on a two-dimensional checkerboard with two-fold dilutions. The final concentrations were 0.002 to 0.5 µg/mL and 1 to 64 µg/mL for isavuconazole and colistin, respectively. Fifty microliters of each concentration were distributed from row 1 to 8 for colistin and from column 1 to 11 for isavuconazole. Column 12 was used a as growth control and contained 100 µL of double strength RPMI medium with 1% of DMSO.

### Inoculum preparation and inoculation of the microplates

Before the preparation of the inoculum, all isolates were subcultured for a second time on Sabouraud agar slants supplemented with chloramphenicol and cycloheximide and incubated at 35 °C, 95% humidity for 24 h. A few colonies were transferred to a sterile tube containing sterile distilled water. The suspension was counted in a haemocytometer and adjusted to 3.5 × 10^6^ CFU/mL in sterile distilled water. This solution was used for the inoculation of the RPMI agar plates. The solution was further diluted to 2 × 10^5^ CFU/mL, and 100 µL of the final inoculum were distributed in each well to inoculate the microdilution plates. The inoculum was further diluted and 100 µL were spread on a Sabouraud dextrose agar plate with a sterile Drigalski spatula. After an incubation time of 24–48 h at 35 °C, the CFU were counted to ensure the inoculum size and the viability of the yeasts. The microplates were incubated at 35 °C, 95% humidity, and the optical densities were read spectrophotometrically at 24 h at a wavelength of 530 nm with the spectrometer MultiSkan FC (Thermo Fisher Scientific). Before the reading, the microplates were shaken with the PMS-1000 Microplate Shaker (Grant Instruments, Shepreth, United Kingdom). In each set of experiments a blank microplate was included. Each well of the blank microplates was inoculated with 100 µL of sterile distilled water. Blank microplates were also incubated at 35 °C, 95% humidity for 24 h. Before calculation of the MICs, the blank plate was subtracted from the microplates inoculated with the yeasts. Experiments were run in triplicate on different days.

### Interpretation of the checkerboard results

#### Fractional inhibition concentration index

The MICs alone and in combination were defined as the lowest concentration that gave 50% of inhibition compared to the growth control determined by spectrophotometric reading. For the calculation of the FICI, the high off-scale MICs were converted to the next log_2_ dilution. The FICI was calculated as follows: FICI = (MIC_in combination_/MIC_alone_)_isavuconazole_ + (MIC_in combination_/MIC_alone_)_colistin_. The FICI data was interpreted following way: FICI ≤ 0.5 = synergy, FICI > 0.5–4 = no interaction, and FICI > 4.0 = antagonism^47^.

#### Response surface analysis

Response surface analysis are independent of endpoint definitions like the FICI analysis. The experimental data generated with the microplates as a percentage of growth compared to the growth control is transformed into a dose–response curve for each drug alone. Based on the Bliss model, a theoretical response surface of the combination corresponding to an indifferent interaction is calculated using the dose–response curves of both drugs. For the calculation of the synergy distribution, the modelled response surface is compared with experimental data. To visualize the results, the synergy levels were mapped onto the experimental combination dose–response surface^48^. To summarize the synergy distribution, the SUM-SYN-ANT metric was used, which represents the sum of synergy and antagonism observed. For the interpretation of the metric, a threshold of 24.5% was used as previously determined^11^. Synergy was assumed when the SUM-SYN-ANT was > 24.5%, and antagonism was assumed when and < − 24.5%. Between − 24.5 and 24.5%, no interaction was concluded. For the calculation of the SUM-SYN-ANT of the different isolates, the results of two different runs have been combined. All calculations were performed using Combenefit software^38^.

#### Preparation of the RPMI agar plates

Fifteen grams of agar were dissolved in 450 mL of distilled water. After the pH was adjusted to 7 with 0.1 M NaOH, the solution was set to 500 mL with distilled water, and autoclaved for 20 min at 121 °C and 2 bars. The liquid agar and 500 mL of double strength RPMI medium were put into a water bath at 50 °C. After the adjustment of the temperature to 50 °C, both solutions were mixed under a flow bench and stirred by a stir fish. Additionally, colistin RPMI agar plates contained the adequate volume of sterile colistin stock solution to a final of 64 µg/mL. Twenty microliters of the liquid RPMI agar or colistin RPMI agar were added to sterile petri dishes (diameter 90 mm) (Merck) under a flow bench and allowed to cool down.

#### Inoculation of RPMI agar plates

The RPMI agar plates were inoculated using the inoculator retro C80 (bioMérieux, Nürtingen, Germany). Briefly, a sterile cotton swap was soaked in the yeast suspension set to 3.5 × 10^6^ CFU/mL, and excess fluid was removed by pressing the swab against the wall of the tube. A cross was gently drawn over the agar surface using the wet cotton swap. After the inoculator was turned on with maximum rotation, the cotton swap was moved from the edge of the agar surface to the middle of plate and back applying a slight pressure. After inoculation, the plates were allowed to dry and isavuconazole gradient concentration strips were placed onto the agar surface. For the activity of isavuconazole alone RPMI agar plates were used. For the activity of colistin alone, and for the combination, colistin RPMI agar plates were used. Activity of colistin alone, was tested by inoculation of the agar plate, without placement of a gradient concentration strip onto the surface. Agar plates were incubated at 35 °C and pictures were taken at 48 h. Reading of the MICs was done according to the recommendations of the manufacturer.

#### Interpretation of the results of RPMI agar plates

A decrease or an increase of ≥ 2 log_2_ dilutions in MIC compared to the most active drug has been defined as synergy or antagonism, respectively. A decrease or an increase of less than 2 log_2_ dilutions in MIC to the most active drug has been defined as no interaction^9^.

#### Comparison of EUCAST and gradient concentration strip MICs

Isavuconazole MICs obtained by EUCAST and gradient concentration strips were compared by a non-parametric unpaired t-test (Mann–Whitney). Essential agreement between the two methods was evaluated by calculating the percentage of the MICs, which differed by no more than 2 log_2_ dilutions.

### Conference presentation

The results of this work (abstract number 2614) were published in the abstract book of the 30th European Congress of Clinical Microbiology and Infectious Diseases, supposed to be held 18th–21st April 2020 in Paris, France, but cancelled due to the COVID-19 pandemic.
